# A hybrid machine learning and explainable AI framework for optimizing risk-based authentication

**DOI:** 10.1371/journal.pone.0349095

**Published:** 2026-05-26

**Authors:** K. Sasikumar, Sivakumar Nagarajan

**Affiliations:** School of Computer Science and Engineering, Vellore Institute of Technology, Vellore, India; University of Kerbala, IRAQ

## Abstract

As online platforms continue to grow, the need for strong authentication mechanisms becomes increasingly important to protect sensitive information and networks. Risk-Based Authentication (RBA) is an adaptive approach that dynamically adjusts authentication decisions based on user behavior and contextual information, thereby improving both security and user experience. This study proposes a hybrid RBA framework that integrates machine learning ensemble techniques, fuzzy logic, clustering, and optimization to enhance account takeover detection and dynamic risk assessment. The ensemble classifier, combining Gradient Boosting, SVM, and XGBoost, predicts the probability of account compromise based on login behavior, device attributes, and network information. K-Means clustering is used to generate initial risk thresholds (low, medium, and high), which are further refined using a fuzzy logic system to map probabilities to risk levels. The L-BFGS-B optimization algorithm is employed to fine-tune fuzzy membership boundaries and improve threshold consistency. Experimental results demonstrate strong performance, achieving 97.77% accuracy, 99.41% precision, 98.04% recall, 98.72% F1-score, and an EER of 0.0303. On large-scale datasets ranging from 2M to 30M records, the proposed framework demonstrates consistent improvement in authentication decisions. For the 2M dataset, Allow Login actions increase from 349,432–349,923, while Deny Login actions decrease from 1,462–1,228, along with a slight reduction in additional authentication prompts. Furthermore, the use of Explainable AI techniques, particularly SHAP, enhances the transparency and interpretability of the model, supporting more informed decision-making. Overall, the proposed framework is accurate, adaptive, and suitable for real-world risk-based authentication applications.

## 1 Introduction

Data security has become the most critical step in today’s digital world to protect data from unauthorized access and prevent security breaches. The widening gap in sensitive information exposure, in the form of both personal and business data, is mainly caused by the rapid adoption of cloud services, mobile devices, and networks that communicate with one another. The use of four fundamental policies-encryption, multifactor authentication (MFA), secure communication, and data management-has been widely acknowledged as the most effective way to ensure data safety. Personal data attacks, such as those that target bank details and medical records, are on the rise. Companies that do not have proper security measures in place are facing identity theft, financial losses, damage to their brand, as disruption in their operations. Moreover, the cost of data breaches is still rising [1,2].

At the global level, the average cost of a data breach was USD 4.88 million in 2024, which is 10% higher than the cost in 2023 [3]. This increase in negative incidents is due to the use of more sophisticated methods and tools for protecting information, as well as to an increase in cyber threats. Strong authentication is needed because it ensures that the user is indeed who they claim to be thus, no one else can access sensitive data, and the organization’s digital assets are safeguarded. State of the art authentication methods have been evolving, however, recent security incidents have shown that inadequate authentication practices are the main reason for data breaches. The auditing of authentication transactions is imperative for security. Authentication records consisting of timestamps, login IP addresses, failed logins, and successful logins provide a wealth of information about user behavior, it is up to the companies to make use of it for strengthening their security, preventing breaches by noticing suspicious activities in their logs, and so forth. Continuous monitoring and meticulous responses to these data streams constitute the bedrock of a strong defense against unauthorized access [4].

**Fig 1:**
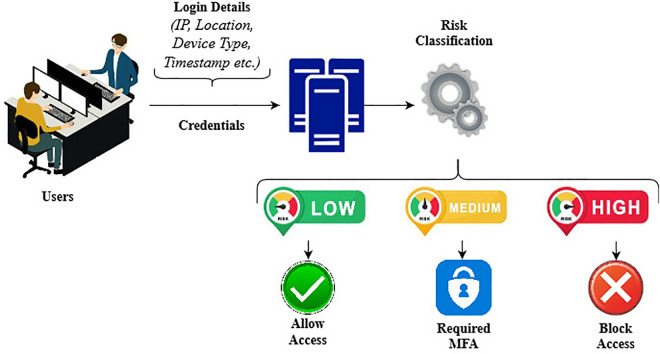
Risk-based authentication.

RBA, as shown in (Fig 1), is an authentication method that offers strong security and the flexibility of a secure environment, thus enhancing password security against various threats. The main threats to RBA are credential stuffing, password guessing and phishing. RBA is a technology that uses additional factors during login and immediately requests another authentication if the user’s login differs from the usual pattern. This method sets authentication rules according to the risk category of the login attempt and considers factors, like location, device type, time of login, and user behavior. The users are permitted to input their password only for activities that are low risk and high-risk activities can call for an MFA and other authentication methods. In addition to providing the required protection, this policy ensure a smoother user experience in the case of low-risk logins. Severe precautions are applied in high-risk cases to ensure quicker and more secure routine access. RBA is applied in areas such as banking, e-commerce, and enterprise systems where a combination of security and user-friendliness is preferred [5,6].

Security levels must be adjusted based on the user activity to identify any suspicious activities that could indicate a security threat. This method gives very strong protection to high-risk operations, demands quieter alerts, and offers a better experience to the real user. Its flexibility lessens the impact of interruptions on users and provides in the required security. Overall, this is a good combination of ease and security. Moreover, the RBA decision-making process should be clear to justify the classification of certain behaviors as risky. Both upstream and downstream users should be aware of the arguments supporting the application of security measures in RBA systems, which alter authentication conditions according to the assessed risk, especially when unusual behavior is detected [7,8]. Recent studies have suggested that current methods for detecting anomalies in logs and RBA models do not ensure user authentication and management security. These systems must deal with the problem of setting thresholds and accurately interpreting the results. The objectives of this study were as follows:

1. **Hybrid Ensemble-Based Risk Prediction:** Created an inclusive RBA model that integrated Gradient Boosting, SVM, and XGBoost for the prediction of account takeover risks.
2. **Dynamic risk thresholding:** Implemented K-means clustering and fuzzy logic to establish dynamic risk thresholds of low, moderate, and high.
3. **Optimized Fuzzy Membership:** Applied L-BFGS-B to the enhancement of fuzzy membership functions for the purpose of dependable and consistent making of decisions.
4. **Interpretable Security Decisions:** Embedding Explainable AI Methods to increase the interpretability of the model

## 2 Related work

This study further develops the anomaly detection by analyzing various techniques, algorithms, and frameworks that can cope with problems associated with complex systems [9]. Consequently, a number of the contemporary models for log anomaly detection still have dependence on a comprehension of the normal system operations. To remedy this scenario, Markus et al. [10] proposed a novel approach that exploits the semantic likenesses present in the log data of different systems thereby lessening the need for comprehensive prior knowledge and enabling the utilization of labeled data in the training phase. Their method combines ensemble learning with self-attention transformers to capture the semantic representation of log data accurately. However, the assumption of semantic similarity may not hold for all systems. Piotr et al. [11] improved the LogEvent2Vec algorithm by narrowing the analysis window to achieve improved anomaly detection and easier sequence analysis. They replaced word2vec with fastText, excluding the character n-grams. Future work may focus on incorporating administrator feedback and adapting the model to anomaly free data; however, scalability issues remain for large or real-time datasets.

This research paper [12] recommends the application of DBSCAN and K-means techniques for the detection of anomalies in log files. K-means has been shown to be the most effective technique for this purpose, as it detect the sequences of logs by recognizing anomalous patterns with a 97% accuracy rate based on the use of IDF representation and feature extraction. In their research, Islami et al. [13] reported that the use of short-text topic modeling with ensemble techniques for anomaly detection yielded better results than traditional LDA. The GSDMM with the XGBoost pair achieved the highest performance among various ensembles, which also comprised random forest, gradient boosting, and AdaBoost. In addition, Optuna was involved in hyperparameter tuning and in that process, XGBoost parameters were improved thus, its performance increased across the board.

The problem of detecting anomalies in log files has been the subject of research [14], the characteristics of which are dynamic features, very large sizes and unstructured formats. Several techniques for detecting anomalies have been compared based on their efficiency, speed and the extent to which they adapt to different log file types. The methods evaluated included statistical techniques, machine learning and deep learning. Moreover, the research study considered the shortcomings of the present methods, such as the inability to detect rare anomalies, issues with log pattern complexity and the problem of accurately interpreting outcomes. Corbelle et al. [15] proposed a log compaction technique to minimize the size and processing time, as well as a hierarchical classification system for detecting anomalies in log data using BERT. They applied cosine similarity to analyze the logs pre- and post-processing. However, the method performs well only when the hierarchical codes are exact; it may struggle when faced with variable log vocabularies. although it enhances efficiency, large log management still requires substantial resources which is a problem for real-time applications. In addition, there are concerns regarding overfitting and generalization.

Landauer et al. [4] reported the results of their survey, which ranked various model architectures, such as RNNs, CNNs, transformers, and unsupervised models, such as autoencoders and GANs, for the purpose of detecting abnormalities. Anomalies can be detected via classification methods or by computing anomaly scores. However, there are many challenges ahead, such as the detection of non-sequential anomalies, lack of model interpretability, limited evaluation dataset diversity, and issue with result reproducibility. Sepczuk et al. [16] suggested a risk analysis and multi-level authentication scheme that offered a compromise between user satisfaction and security. It revolves around context-aware secure authentication, however, questions regarding its applicability have arisen because of insufficient validation across different scenarios. In a collaborative effort, Anvesh et al. [6] supplied a literature survey of RBA with Ping Risk, highlighting its importance in cybersecurity, that is, user-friendly adaptive authentication. Ping Risk leverages advanced algorithms to conduct real-time risk assessments and monitoring which makes it possible to identify dangers promptly and, hence, increases the security level.

Charmet et al. [17] noted that the application of artificial intelligence in security devices often led to their incapacity to communicate results in a simple manner, which once again resulted in human misunderstanding. XAI is the result of this problem, as it is aimed at making AI models more transparent. The authors performed a thorough survey of research conducted at the crossroads of XAI and cybersecurity, especially in the areas of intrusion detection and malware classification. In addition, they mentioned the security problems initiated by XAI, such as the insecurity of XAI systems against attacks and some of the strategies to counter them.

Gencer et al. [18] introduced a fuzzy Common Vulnerability Scoring System (CVSS) that integrates the cyber security risk evaluation process using fuzzy logistic regression and least-squares estimation. The model fuses traditional CVSS with fuzzy and linguistic elements to offer greater adaptability and precision. The classification is improved as the numerical inputs are converted into fuzzy outputs, resulting in less ambiguity in the risk assessment. The mean squared error (MSE) and mean absolute error (MAE) were utilized to evaluate the model’s performance and demonstrated to surpass traditional approaches. In a similar study, Zhou et al. [17] used LLMs and BERT for log anomaly detection which not only enhanced the understanding of meanings but also the accuracy of detection compared to rule-based methods. The method proposed scored higher than the typical techniques with precision and recall rates of 90% or high. This method demonstrate the possibility of RBA improvement through advanced log analysis.

Existing techniques for identifying suspicious logs and RBAs have several major limitations, such as dependentce on previously defined system behavior, inability to process large data sets, imposition of fixed risk levels, and lack of explanation. The use of machine learning will allow for the real-time detection of patterns, and the application of clustering for threshold optimization can enhance the scalability of the system, whereas setting of dynamic authentication thresholds can eliminate of false positives/negatives. Moreover, XAI is a crucial factor in the perception of authentication decisions, as it makes it possible for IT personnel to be aware of the risk levels and to properly modify the security policy. Our method effectively addresses these problems by increasing the detection rate, adaptability, interpretability, and transparency of the decision-making process for risk-based authentication.

### 2.1 Structure of the paper

The remainder of this study is structured as follows. In Section 2, the proposed approach is presented along with the data processing, machine learning techniques, clustering, fuzzy logic, and threshold optimization. In Section 3, the results of the experiments are evaluated, and their performances are compared with those of the baseline models. In Section 4, model explainability is discussed with reference to explainable AI (XAI), and Section 5 concludes with a summary of the important findings and suggestions for future research.

## 3 Proposed technique

In this study, we present the creation of a machine learning pipeline for the identification of account takeover attacks using an ensemble-based method. The first step is data pre-processing, which consists of the extraction of time-based features, normalization of numerical aspects, and encoding of categorical variables. We chose machine learning-based methods as the baseline models mainly because of their effectiveness and interpretability, which are the two factors that make them more appropriate for real-time authentication than deep learning models, which demand massive computational power and large labeled datasets. The machine learning pipeline is built around three classifiers: gradient boosting, support vector machine (SVM), and XGBoost, which are integrated through a soft voting classifier that combines the predicted probabilities from each model to make the final decision. Furthermore, the pipeline incorporates a receiver operating characteristic (ROC) curve to optimize the decision thresholds, which are then adjusted using clustering techniques to classify the risk levels as low, medium, or high.

**Fig 2:**
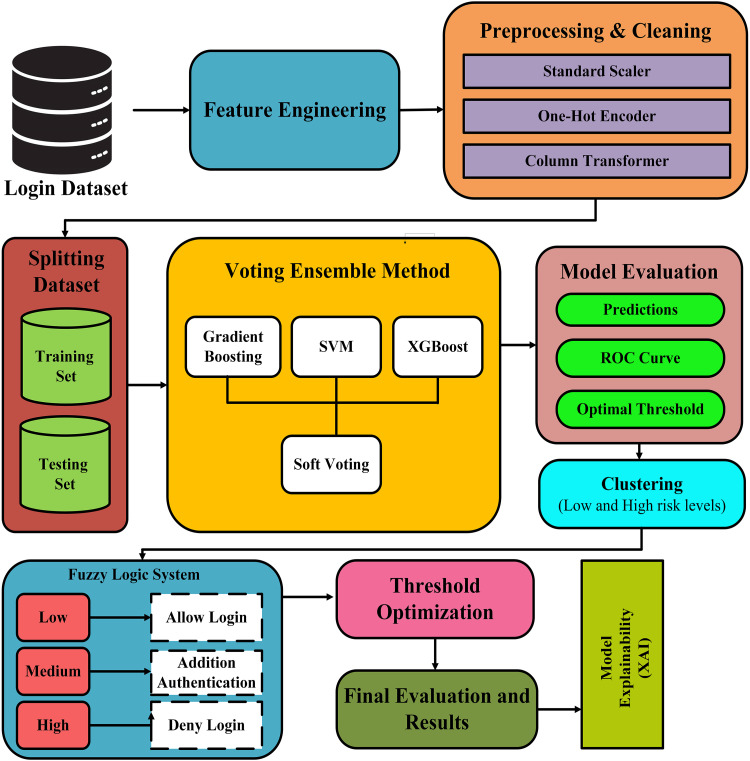
Architecture of proposed technique.

A fuzzy logic system is used to transform probabilities into related risk levels and suggested actions, such as allowing a login, requesting further additional authentication, or denying login attempts. This method attempts to increase the interpretability of the model and simplify decision-making. Gradient-based algorithms have been used to fine-tune the threshold value to improve the classification accuracy. XAI was used to enhance the comprehension of the results. (Fig 2) depicts the architecture of the methods used in this study. The stages of this study are as follows:-

### 3.1 Dataset description

We used a login dataset [19,20] that included 15 attributes and synthesized feature data from 33 million login attempts made by 3.3 million users. The purpose of this dataset is to further develop RBA system research and development. It was produced using actual login activity observed in a major Norwegian single sign-on (SSO) service. The properties of these datasets are listed in Table 1. of the 33 million records, we chose a random subsect of 2 million for the initial model selection step to ensure a representative sample of different login behaviors and attack patterns. This enabled us to perform an efficient comparison of the machine learning models in terms of precision, recall, and F1-score. The best-performing model was then selected, and we trained and evaluated it with the 30M dataset to verify the strength of its performance and suitability for practical use. Thus, we achieved a compromise between a computationally intensive process and a thorough examination of the model.

**Table 1:** Dataset attribute description details.

Feature	Description
IP Address	IP address associated with the attempted login.
Country	Country associated with the IP address.
Region	Region information derived from the IP address.
City	City identified based on the IP address.
ASN	Autonomous system number generated based on the IP address.
User Agent String	User agent string provided by the client.
OS Name and Version	Operating system name and version parsed from the user agent string.
Device Type	Type of device determined from the user agent string.
User ID	Unique identifier associated with the affected user account.
Round-Trip Time [ms]	Client–server delay measured from the server side.
Login Successful	Indicates whether the login attempt succeeded (True/False).
Is Attack IP	Indicates whether the IP address appears in a known attacker database.
Is Account Takeover	Login attempt flagged by incident response as account takeover.

The features chosen and their respective roles in detecting account takeovers are as follows: User agent string and device type changes indicate a device or browser switch; operating system and version indicate unexpected OS logins; login time and day of the week indicate out-of-pattern logins; round-trip time refers to network anomalies, such as proxies or VPNs; attack IP points to logins from malicious IPs; and Account Takeover, as the target variable, enables the model to learn from past compromises.

### 3.2 Exploratory data analysis

**Fig 3:**
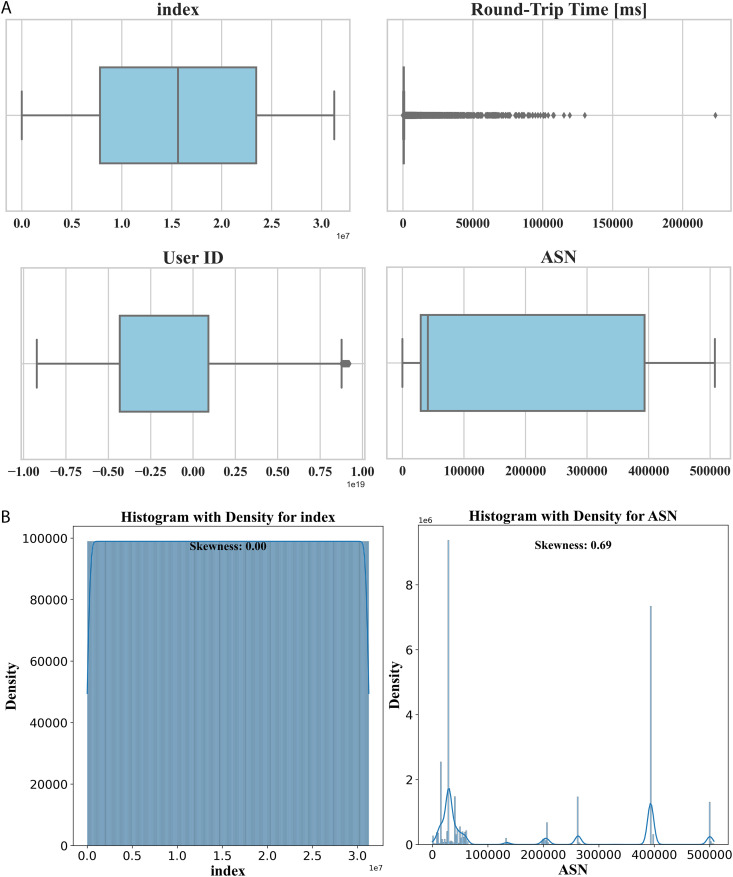
(a) shows box plots that help visually detect outliers, while (b) demonstrates addressing skewness by applying a log transformation.

The first step in the ML model building process is dataset analysis, which aims to identify the main characteristics of the dataset, and is usually performed visual methods. This step is significant because it prepares the model by showing the data distribution. It is important to examine skewness, outliers, and the data distribution in general. For features that were highly skewed, a log transformation was performed, which resulted in data that were more suitable for modeling. This method is indeed very much effective in feature engineering and preprocessing as it helps in making the data properly organized and in the right condition for analysis. The (Figs 3a and 3b) portray this process.

### 3.3 Feature engineering

The main login patterns were identified using time-based feature extraction. The feature login_hour indicates the time of login, which is helpful in identifying strange login times. The feature login_day_of_week tells which day of the week it is, so one can analyze user activity during the week and the weekend. Additionally, the feature login_is_weekend generates a binary signal for weekend logins, hence, it gives an idea of login patterns during non-working days. All these features have a positive influence on the model’s ability to detect irregularities in login activity.

### 3.4 Data preprocessing and splitting

At this point, we direct our attention to the process of feature extraction, which takes place in two steps: one for the numerical and the other for the categorical features. The numerical features of this model are ASN, login_hour, and login_day_of_week, while the categorical features are country, Region, City, user agent string, OS name and version, device type, and login_is_weekend. To make the numerical features suitable for mixing with the model, we used the standard scalar method for scaling, which means that the features were changed to have a mean of zero and a standard deviation of one. However, this scaling is vital mainly for building blocks that rely on the size of the feature, as it guarantees that no numeric feature is favored or treated differently than others. For the categorical attributes, we used one-hot encoding, and every category was turned into a binary matrix where all categories created a column of ones (for the existing category) and zeros (for others). The Column Transformer executed these conversions, and we simultaneously scaled the numeric features and encoded the categorical features. This combined strategy means that all preprocessing steps take place uniformly in the training and testing datasets, thus preserving the data integrity throughout the process [21]. First, the login dataset was divided using a stratified sampling method where the training and test sets were created by applying a train_test_split with an 80−20 ratio between them. Stratification was implemented to maintain the same class proportions in both sets.

### 3.5 ML models

The ensemble model with a Voting Classifier that combined Gradient Boosting, SVM, and XGBoost was used to classify account takeover attempts and normal activities with better accuracy and reliability after preprocessing. Among various machine learning models, XGBoost, and SVM were preferred over deep learning models due to their computational efficiency, good performance with small datasets, ease of interpretation, and faster training time. Moreover, these models are less likely to overfit which is a prominent feature for real-time applications as well as for processing large amounts of data. The SVM was selected for its capability to deal with nonlinear decision boundaries in high-dimensional areas with slight class margins. In addition to being able to work with unbalanced datasets, XGBoost also interacts with the features, deals with missing data, and minimizes overfitting through regularization. Gradient boosting was preferred because of its iterative learning which, in turn, improved accuracy in the case of large datasets with complex patterns. The hyperparameters for each model were as follows: gradient boosting learning rate = 0.1, n_estimators = 100, max_depth = 3; SVM with an RBF kernel, C = 1, and probability estimates; and XGBoost learning rate of 0.1, n_estimators = 100, max_depth = 6, and log loss as the evaluation metric.

#### 3.5.1 Gradient boosting classifier.

In gradient boosting, the model is built sequentially, with each stage contributing a weak learner to solve the errors in the previous models. In each stage *m*, the technique incorporates a new function *h*_*m*_(*x*) into the previous model to minimize the differentiable loss function $L(y,F_{m-1}(x))$.


$$\begin{array}{c} F_{m}(x)=F_{m-1}(x)+\eta h_{m}(x) \end{array}$$ (1)

In Equation 1, *F*_*m*_(*x*) denotes the model’s prediction at stage *m*. The parameter η represents the learning rate, which controls the impact of weak learners on the overall model. The term *h*_*m*_(*x*) refers to a weak learner in stage *m*. The purpose of each new weak learner, denoted by *h*_*m*_(*x*), is to reduce the residual error through training by conforming to the loss function negative gradient $L(y,F_{m-1}(x))$. Using this approach, the model can correct previous errors. Specifically, the model adds learner *h*_*m*_(*x*), which approximates


$$\begin{array}{c} h_{m}(x)=-\nabla_{F_{m-1}}L(y,F_{m-1}(x)) \end{array}$$ (2)

After a specific number of iterations, this procedure produces the final model *F*_*M*_(*x*), which is a weighted combination of all weak learners, as shown in Equation 3 where *M* is the total number of iterations.


$$\begin{array}{c} F_{M}(x)=F_{0}(x)+\eta \sum_{m=1}^{M}h_{m}(x) \end{array}$$ (3)

By progressively incorporating these weak learners, the accuracy of the model is improved by rectifying the earlier errors in each step [22,23].

#### 3.5.2 Support vector machine (SVM).

SVM is a key component of the voting classifier ensemble. SVC seeks to identify the optimum hyperplane for optimizing the margin between classes. When the data cannot be separated linearly, slack variables $\xi_{i}$ are introduced to accommodate misclassifications. The optimization function is defined as follows:


$$\begin{array}{c} \min\frac{1}{2}\|w{\|}^{2}+C\sum_{i=1}^{n}\xi_{i} \end{array}$$ (4)

subject to:


$$\begin{array}{c} y_{i}(w\cdot x_{i}+b)\geq 1-\xi_{i},\;\xi_{i}\geq 0 \end{array}$$ (5)

where *w* is the weight vector for the hyperplane and *C* is the regularization value that optimizes the margin while minimizing the classification error. In this model, the SVM model was calibrated with the probability = true. By doing this, the SVM was able to provide class probabilities instead of just binary predictions. The SVC predictions suitable for soft voting are obtained by applying a logistic regression model to the decision values. The class_weight = ’balanced’ parameter is very advantageous for the model dealing with imbalanced datasets, as it automatically assigns weights to the classes relative to their frequency in the dataset.

The SVM classifier’s probability estimates in the ensemble were combined with those of the Gradient Boosting and XGBoost classifiers using a soft voting technique, which averaged the predicted probabilities. This method takes advantage of SVM’s strength in handling complicated decision boundaries and thus contributes to the total strength of the ensemble. The ensemble method increases the precision and versatility by merging the probability-based forecasts of all the classifiers, which is especially advantageous for imbalanced datasets [24,25].

#### 3.5.3 XGBoost.

XGBoost was used in the ensemble model to make probability estimates, which were then averaged through the predictions of the Gradient Boosting and SVC classifier. XGBoost improves generalization through gradient boosting with combined regularization, as indicated by its objective function in Equation 6.


$$\begin{array}{c} L(\theta )=\sum_{i=1}^{n}l(y_{i},{\hat{y}}_{i})+\sum_{k=1}^{K}\Omega (f_{k}) \end{array}$$ (6)

The log loss function for classification is represented as $l(y_{i},{\hat{y}}_{i})$ and regularization term $\Omega (f_{k})$


$$\begin{array}{c} l(y_{i},{\hat{y}}_{i})=-\left(y_{i}\log({\hat{y}}_{i})+(1-y_{i})\log(1-{\hat{y}}_{i})\right) \end{array}$$ (7)

$$\begin{array}{c} \Omega (f_{k})=\gamma T+\frac{1}{2}\lambda \|w{\|}^{2} \end{array}$$ (8)

Parameter *T* denotes the total number of leaf nodes, γ indicates the penalty for each leaf node, and λ corresponds to the L2 regularization applied to the weights *w*, thereby facilitating the control of the model’s complexity to avoid overfitting [26]. In the final stage of the ensemble model, the predictions of gradient boosting, SVC, and XGBoost are forwarded to the Voting classifier, which combines their outputs. This is through a soft voting strategy, whereby the overall probability for each class is calculated by considering the average of the predicted probabilities from all classifiers involved. A mathematical description of this process is given in Equation 9.


$$\begin{array}{c} P_{\text{ensemble}}(y)=\frac{1}{N}\sum_{i=1}^{N}P_{i}(y) \end{array}$$ (9)

Here, *P*_ensemble_(*y*) is the class probability predicted by the Voting Classifier. *N* = 3 indicates that three classifiers are involved. *P*_*i*_(*y*) represents the probability of class *y* for the *i*-th classifier. By averaging these probabilities, the Voting Classifier exploits the strength of each model to obtain a more reliable and accurate prediction [27].

### 3.6 Model evaluation

Predictions were made by the classifier, and the model’s effectiveness was assessed using several key metrics, such as the classification report and ROC-AUC score [28]. The classification report encompasses accuracy, precision, recall, and F1-score metrics for both weighted and macro averages; further information is provided in Table 2. The ROC-AUC score reflects the area beneath the ROC curve, which is determined using Equation 10, thus indicating the model’s capability of distinguishing between positive and negative classes. An AUC score closer to 1 signifies better performance.


$$\begin{array}{c} \text{AUC}=\int_{0}^{1}\text{TPR}(\text{FPR})\;d(\text{FPR}) \end{array}$$ (10)

**Table 2:** Performance metrics and their mathematical definitions.

Metric	Description	Formula
Accuracy	Measures the overall proportion of correctly classified samples across all classes.	$\text{Accuracy}=\frac{\sum_{i=1}^{C}TP_{i}}{\sum_{i=1}^{C}(TP_{i}+FP_{i}+FN_{i})}$
Precision (Macro)	How many predicted positive samples for each class are correct, averaged across classes.	${\text{Precision}}_{macro}=\frac{1}{C}\sum_{i=1}^{C}\frac{TP_{i}}{TP_{i}+FP_{i}}$
Recall (Macro)	Model’s ability to correctly identify actual positive samples.	${\text{Recall}}_{macro}=\frac{1}{C}\sum_{i=1}^{C}\frac{TP_{i}}{TP_{i}+FN_{i}}$
F1-Score (Macro)	Harmonic mean of precision and recall for each class, averaged.	${\text{F1}}_{macro}=\frac{1}{C}\sum_{i=1}^{C}\frac{2TP_{i}}{2TP_{i}+FP_{i}+FN_{i}}$
Equal Error Rate (EER)	Point where False Positive Rate equals False Negative Rate.	EER=FPR where FPR=1−TPR

To determine the initial medium threshold for classification, the optimal threshold (denoted as $\theta^{*}$) is selected by maximizing the difference between the true positive rate (TPR) and the false positive rate (FPR), known as Youden’s Index. This is mathematically expressed as:


$$\begin{array}{c} \theta^{*}=\arg\max_{\theta }\left(\text{TPR}(\theta )-\text{FPR}(\theta )\right) \end{array}$$ (11)

### 3.7 Clustering

K-means clustering [29] was selected for its simplicity, low computational cost, and ability to handle large-scale datasets. Its centroid-based nature partitions the data into clusters by assigning each data point to the nearest cluster center, thereby minimizing the total intra-cluster variance (sum of squared distances between data points and their respective centroids). This results in clearer separation of data groups and improves classification performance by distinguishing low- and high-risk patterns more effectively. Compared to other clustering techniques, K-means is computationally efficient and well-suited for large datasets, which is essential for the proposed authentication framework. The objective function of K-means clustering is given by:


$$\begin{array}{c} \min\sum_{i=1}^{k}\sum_{x\in S_{i}}\|x-\mu_{i}{\|}^{2} \end{array}$$ (12)

where *S*_*i*_ represents the set of points in the *i*-th cluster, $\mu_{i}$ denotes the centroid of th *i*-th cluster, and *k* is the total number of clusters.

In the proposed framework, K-means clustering is used to refine the low and high thresholds based on predicted probability scores. After determining the medium threshold using ROC analysis, the probability values are divided into two regions: values below the medium threshold and values above it. K-means clustering (with *k* = 2) is then applied separately to each region.

### 3.8 Fuzzy logic system

This study employs fuzzy logic [30] to categorize predicted probabilities into three risk levels: “low,” “medium,” and “high,” thereby enhancing the model’s decision-making abilities. The categories were established using triangular membership functions, where the parameters *a*, *b*, and *c* represent the start, peak, and end points, respectively. The variable *x* denotes the predicted probability.


$$\begin{array}{c} \mu (x;a,b,c)=\left\{ \begin{array}{cc} 0, & x\leq a\\ \frac{x-a}{b-a}, & a<x\leq b\\ \frac{c-x}{c-b}, & b<x\leq c\\ 0, & x>c \end{array}\right. \end{array}$$ (13)

The decision-making process of the system is guided by fuzzy inference rules such as IF; the probability is low; THEN the risk is low; IF the probability is medium, THEN the risk is medium; IF the probability is high; and the risk is high. The min-max approach is employed to merge these rules and assess the strength of each rule’s output. Finally, the centroid method is employed to represent the results for a particular risk value, as indicated in Equation 14, which is subsequently applied to the final decision making process in the classification task.


$$\begin{array}{c} Z=\frac{\int_{\text{Universe}}\mu (z)\;dz}{\int_{\text{Universe}}\mu (z)\cdot z\;dz} \end{array}$$ (14)

### 3.9 Threshold optimization

To improve the classification thresholds, the Limited-memory Broyden–Fletcher–Goldfarb–Shanno with box constraints (L-BFGS-B) [31,32] optimization algorithm is employed. The objective of the optimization is to improve the consistency of authentication decisions by minimizing the difference between initial and updated decisions.


$$\begin{array}{c} \text{Objective}=-\frac{1}{N}\sum_{i=1}^{N}1(a_{i}={\hat{a}}_{i}) \end{array}$$ (15)

where *N* is the total number of instances, *a*_*i*_ represents the initial decision (action), and ${\hat{a}}_{i}$ denotes the updated decision after applying optimized thresholds. The objective function evaluates the consistency between initial and optimized decisions. This formulation ensures that the optimization process refines the thresholds while preserving stable decision behavior. Initial threshold values for low, medium, and high risk are set based on prior analysis. The optimization is performed under box constraints to ensure valid probability ranges:


$$\begin{array}{c} 0\leq \text{low}<\text{medium}<\text{high}\leq 1 \end{array}$$ (16)

The L-BFGS-B algorithm updates the thresholds iteratively using a gradient-based approach:


$$\begin{array}{c} x_{k+1}=x_{k}-\alpha \nabla f(x_{k}) \end{array}$$ (17)

where *x*_*k*_ represents the current threshold values, $\nabla f(x_{k})$ is the gradient of the objective function, and α is the step size. The optimization continues until convergence criteria are met, including minimal change in objective value, minimal change in threshold values, or reaching the maximum number of iterations. The optimized thresholds are then used within the fuzzy logic system to assign final risk levels.

The optimization process continues until one of the following convergence criteria is satisfied:

1. **Convergence in Function Value:** The change in the value of the objective function becomes sufficiently small between iterations.
2. **Convergence in Parameter Values:** The change in the threshold values between iterations becomes sufficiently small.
3. **Maximum Iterations:** Optimization stops after reaching the maximum number of iterations (maxiter = 20) if convergence is not achieved. The algorithm also terminates if the change in threshold values falls below the tolerance value ($gtol=1\times 10^{-6}$).

Once the algorithm converges, the optimized threshold values are obtained as opt_thresholds=result.x. These thresholds represent the values that improve the consistency of authentication decisions. After optimization, the refined thresholds are integrated into the fuzzy logic system, which assigns risk levels based on the optimized threshold values.

## 4 Experimental results

This section presents and analyzes the experimental results. ML models were developed using Scikit-learn, and experiments were conducted in Jupyter. The effectiveness of the ensemble model was evaluated against that of the other models, and threshold values were determined using both ROC and ROC combined with clustering to detect differences in action counts. Various optimization methods were employed and compared with alternative strategies, with action counts recorded before and after the optimization process. In addition, the performance of the models was evaluated for different dataset sizes.

### 4.1 ML and DL Models

**Fig 4:**
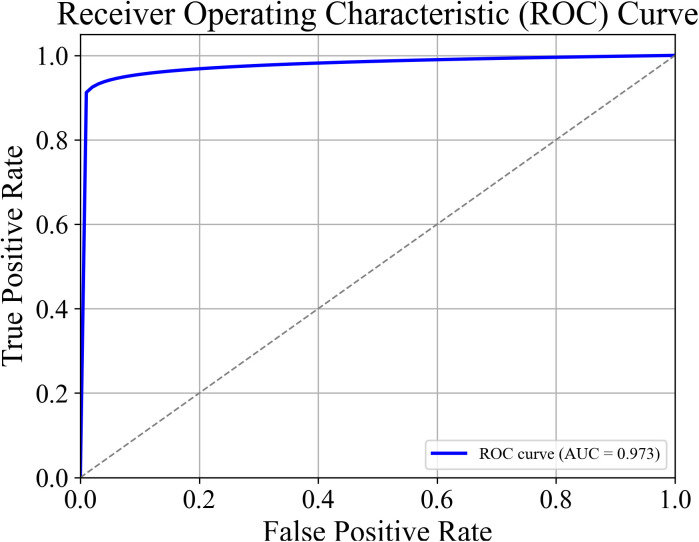
ROC cure for ensemble model for 2M dataset size.

This study evaluates the performance of different machine learning models, including Gradient Boosting (GB), Random Forest (RF), Support Vector Machine (SVM), and XGBoost (XGB), along with ensemble and deep learning approaches, using six metrics: accuracy, precision, recall, F1-score, MCC, and EER. The results shown in Table 3 highlight the differences between models in terms of classification performance and authentication effectiveness. For example, XGB (81.87% accuracy, 82.2% precision) and SVM (84.1% accuracy, 84.1% precision) show moderate performance, while GB (84.3% accuracy, 85.1% precision) produces relatively more false positives. To improve performance, ensemble methods are applied. Among them, the Soft Voting model (GB + SVM + XGB) performs the best, achieving 97.77% accuracy, 99.41% precision, 98.04% recall, and 98.72% F1-score. It also has the highest MCC (0.9035) and the lowest EER (0.0303), showing better reliability. The ROC curve in (Fig 4) further supports its strong performance.

**Table 3:** Model performance comparison (results calculated for 2M dataset size).

Model	Accuracy	Precision	Recall	F1-Score	MCC	EER
XGB	81.87	82.2	78.4	80.26	0.636	0.183
Random Forest	81.6	82.7	81.6	81.0	0.631	0.184
SVM	84.1	84.1	83.0	84.1	0.690	0.1553
GB	84.3	85.1	84.0	84.0	0.699	0.1507
Hard Voting (GB + SVM + XGB)	89.46	91.0	89.0	89.99	0.789	0.1051
Boosting (GB + Ada Boost + XGB)	93.19	94.9	94.3	94.60	0.854	0.0721
Bagging (RF + SVM + ETC)	93.49	95.2	94.8	94.9	0.857	0.0709
Stacking (GB + SVM + XGB, MC = RF)	96.7	98.6	97.7	98.15	0.910	0.025
Soft Voting (GB + RF + XGB)	94.99	96.2	95.0	95.6	0.8979	0.0501
Soft Voting (LGBM + SVM + XGB)	94.81	96.99	96.50	96.75	0.890	0.040
MLP (Deep Learning)	94.8	95.6	94.2	94.9	0.88	0.045
LSTM (Deep Learning)	93.9	94.8	93.1	93.9	0.87	0.050
**Soft Voting (GB + SVM + XGB)**	**97.77**	**99.41**	**98.04**	**98.72**	**0.9035**	**0.0303**

In comparison, deep learning models such as MLP and LSTM also perform well, with accuracies of 94.8% and 93.9%, respectively. However, their performance is still lower than the proposed ensemble model, especially in terms of MCC and EER. Other ensemble methods also show slightly lower performance. Overall, the Soft Voting (GB + SVM + XGB) model gives the best results across all metrics and is the most suitable choice for risk-based authentication.

To further assess the stability of the proposed model and examine potential overfitting, repeated runs and cross-validation were performed. The model achieved an accuracy of 97.77% ± 0.21 across repeated runs. In addition, 5-fold cross-validation resulted in a mean accuracy of 97.65% with a standard deviation of 0.32, and a 95% confidence interval of [97.20%, 98.10%]. These results indicate consistent performance with low variance, suggesting that the model maintains stable performance and is not overly fitted to the dataset.

### 4.2 Threshold and action count analysis for ensemble model

**Fig 5:**
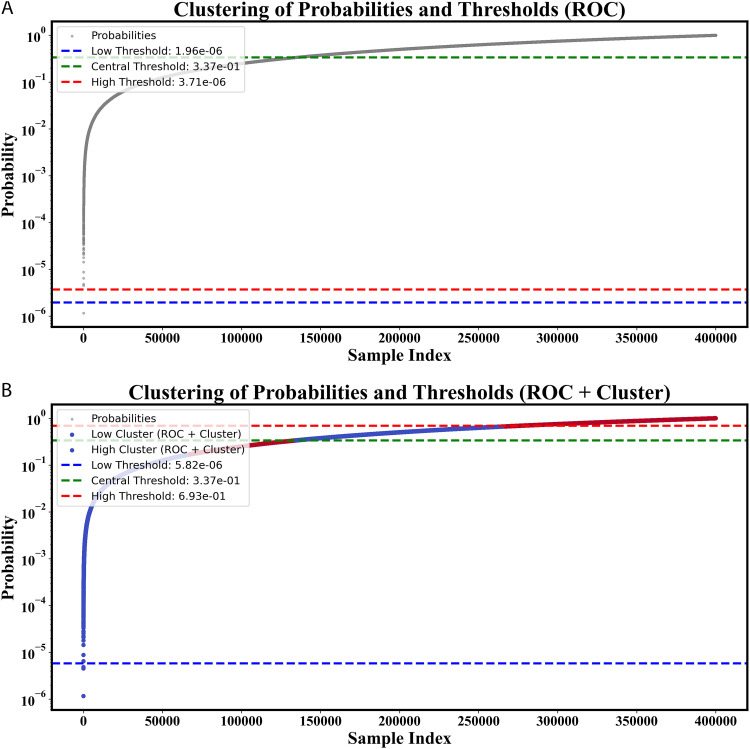
(a) Displays the thresholds from the ROC method before clustering, where (b) shows probability clustering using ROC  +  Cluster, which improves the classification boundaries for the 2M dataset.

The ensemble model showed a significant improvement when fuzzy logic was applied to the ROC + clustering thresholds. The low threshold increased significantly from 1.9601*e*^−06^ (ROC) to 5.8246*e*^−06^ (ROC + Clustering), the medium threshold remained the same at 0.3371, and the high threshold increased to 0.6928. Probability clustering visualizations are shown in (Fig 5).

First, the model was able to process 89,798 allowed login actions, after which 208,335 actions were tagged for extra authentication and 101,867 actions were without login. The combination of ROC + clustering and fuzzy logic brought about improvement, which is reflected in Table 4, and the new figures were 349,432 low-login actions, 48,132 requests for additional authentication, and 2,436 denying login actions. The results demonstrate that the ensemble model is very effective at improving the decision process through threshold adjustment, thus making it a reliable option for this application.

**Table 4:** Consolidated table for threshold values and action counts (2M dataset).

Models	Threshold Value (ROC)			Threshold Value (ROC + Cluster)			Action Count (ROC Threshold)			Action Count (ROC + Cluster Threshold)		
	Low	Med	High	Low	Med	High	Allow	Req.Auth	Deny	Allow	Req.Auth	Deny
Gradient Boosting	2.8128*e*^−06^	1.0	1.25	3.7505*e*^−06^	1.0	1.0	1	399999	0	348805	49254	1941
Random Forest	0.0125	0.19	0.4826	1.7251*e*^−06^	0.1400	0.6899	399964	30	6	349271	48540	2189
SVM	1.8618*e*^−06^	0.0002	6.5778*e*^−06^	4.2231*e*^−06^	0.0002	0.3063	92150	205877	101973	350597	47157	2246
XGBoost	1.2442*e*^−07^	0.0060	1.5550*e*^−06^	1.2539*e*^−06^	0.0060	0.7577	181478	180884	37638	348988	48331	2671
GB + SVM + XGB (SV)	1.9601*e*^−06^	0.3371	3.7066*e*^−06^	5.8246*e*^−06^	0.3371	0.6928	89798	208335	101867	349432	48132	2436

### 4.3 Threshold optimization

**Fig 6:**
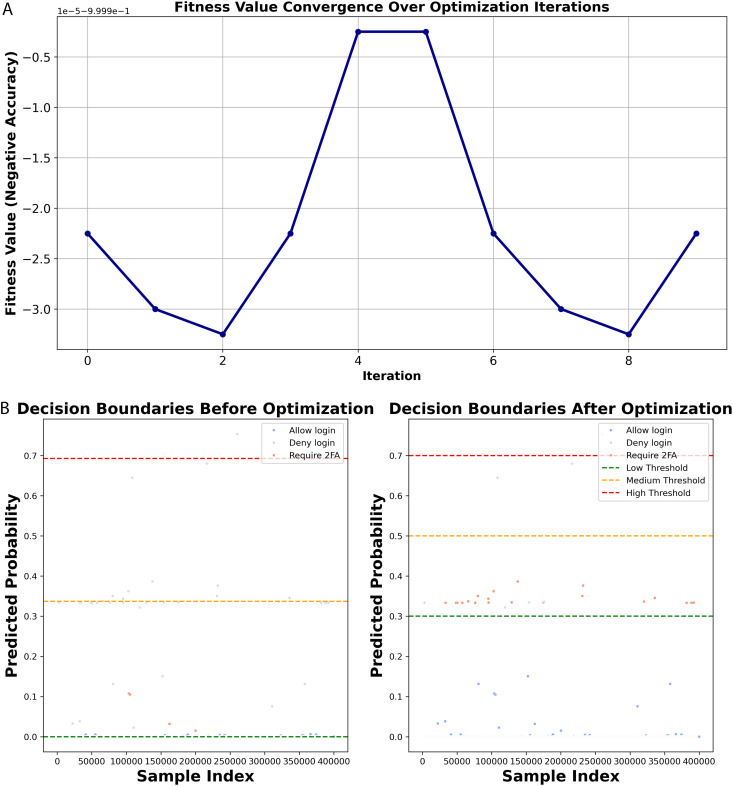
(a) illustrates the convergence of the fitness values, whereas (b) depicts the decision boundaries before and after optimization for the 2M dataset size using L-BFGS-B.

In the case of the ensemble method, we utilized several optimization strategies, namely PSO, Bayesian Optimization, CMA-ES, Simulated Annealing, and Gradient-based optimization (L-BFGS-B). PSO made a significant difference in the results for the low and high thresholds, however, Bayesian optimization and CMA-ES were more conservative in their impact on the changes. Simulated annealing provided balanced adjustments, and L-BFGS-B produced stable thresholds, particularly at medium and high values. (Fig 6) illustrates the convergence of fitness values throughout the optimization iterations, as well as the decision boundaries before and after optimization using L-BFGS-B for the 2M dataset size. The L-BFGS-B optimizer proved to be the most effective for fine-tuning the threshold values and improving decision-making. Initially, the low-, medium-, and high-risk thresholds were 5.8246e^−06^, 0.3371, and 0.6928, respectively. After the optimization process, the values for the decision boundaries were set to 0.3000, 0.5000, and 0.7000 respectively, to provide more distinct decision boundaries, as shown in Table 5. L-BFGS-B yielded the most distinct thresholds in contrast with other methods, where the thresholds resulteing from PSO (0.0000, 0.3277, 0.9000), Bayesian (0.0017, 0.1026, 0.2324), CMA-ES (0.0076, 0.1138, 0.5655), and simulated annealing (0.0264, 0.2284, 0.3617) overlappe or were inconsistent, thus leading to less reliable risk classification.

**Table 5:** Threshold values and action counts before and after optimization for ensemble method (2M dataset).

Optimizer	Threshold Before Opt.			Threshold After Opt.			Action Count Before Opt.			Action Count After Opt.		
	Low	Med	High	Low	Med	High	Allow	Req.Auth	Deny	Allow	Req.Auth	Deny
PSO	5.8246*e*^−06^	0.3371	0.6928	0.0000	0.3277	0.9000	349432	48132	2436	349432	49106	1462
Bayesian	5.8246*e*^−06^	0.3371	0.6928	0.0017	0.1026	0.2324	349432	48132	2436	349432	49106	1462
CMA-ES	5.8246*e*^−06^	0.3371	0.6928	0.0076	0.1138	0.5655	349432	48132	2436	349596	48455	1462
Simulated Annealing	5.8246*e*^−06^	0.3371	0.6928	0.0264	0.2284	0.3617	349432	48132	2436	351068	46983	1949
L-BFGS-B	5.8246*e*^−06^	0.3371	0.6928	0.3000	0.5000	0.7000	349432	48132	2436	349923	48849	1228

Furthermore, the L-BFGS-B method that was used for optimization brought about notable changes in the authentication actions, particularly the allowed logins which were increased to 349923, the extra login requests which were minimized to 48849, and the cases of Deny Login which were rightly calibrated to 1228, thus paving the way for a good trade-off between security and user-friendliness as depicted in Table 5. On the contrary, the other methods like PSO, Bayesian, and CMA-ES, merely maintained 349432 logins, 49106 additional authentication requests, and 1462 denials, indicating that they had less room for flexibility in the improvement of security actions. Simulated Annealing made some improvements, but it was still less effective than L-BFGS-B, which offered the best balance between security and usability. The distinct threshold separation, the optimized authentication actions, and the refined risk classification, which are shown in Table 5, all lead to the conclusion that L-BFGS-B is the most effective of the optimization methods used in this research.

**Fig 7:**
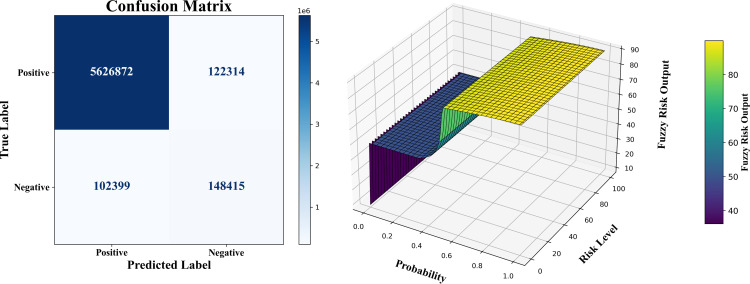
30M dataset size performance: Confusion matrix & fuzzy output.

**Fig 8:**
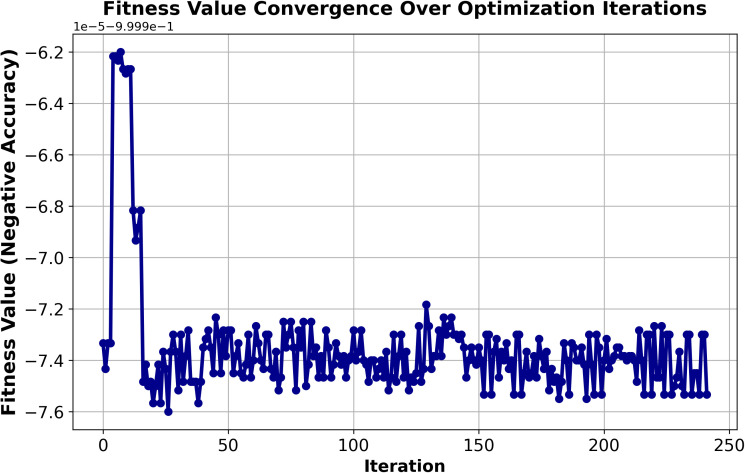
30M dataset size performance: Fitness convergence.

**Fig 9:**
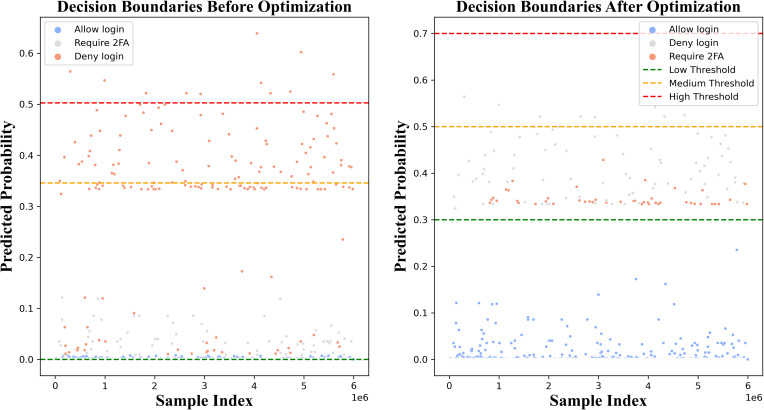
Comparison of decision boundaries before and after optimization for 30 million dataset Size.

The performance of the proposed ensemble model combined with fuzzy logic and L-BFGS-B optimization is presented in Table 6 across varying dataset sizes ranging from 2M to 30M records. The results indicate that, after optimization, significant improvements in accuracy and precision are achieved. The Soft Voting (GB + SVM + XGB) model achieves the highest accuracy of 97.77% on the 2M dataset, while the accuracy ranges from 79.9% on the 4M dataset to a peak of 98.14% on the 20M dataset. Precision remains consistently high, reaching a maximum of 99.41% for the 2M dataset. After optimization, threshold values for low, medium, and high-risk categories are adjusted, resulting in more reliable decision boundaries. Consequently, the counts of Allow Login and Deny Login actions improve, reflecting enhanced classification and decision-making performance. As a specific example, for the 30M dataset, the number of Allow Login actions increases from 5,504,412–5,705,847, while Deny Login actions decrease from 29,807–9,875 after optimization. (Figs 7–9) illustrate the overall results for the 30M dataset.

**Table 6:** Data size, accuracy, precision, recall, F1-score and optimization results.

Data Size	Accuracy	Precision	Recall	F1-Score	Threshold Before Opt.			Threshold After Opt.			Action Count Before Opt.			Action Count After Opt.		
					Low	Medium	High	Low	Medium	High	Allow	Req. add. Auth	Deny	Allow	Req. add. Auth	Deny
2M	97.77%	99.41%	98.04%	98.72%	5.8246*e*−06	0.3371	0.6928	0.3	0.5	0.7	349432	49106	1462	349923	48849	1228
4M	79.9%	92.9%	83.7%	88.1%	9.9483*e*−07	3.1223*e*−06	0.3625	0.2999	0.5	0.7	737655	60002	2343	738855	59200	1945
6M	96.32%	97.41%	96.40%	96.40%	8.0587*e*−07	3.7916*e*−06	0.3730	0.3	0.5	0.7	1082523	111665	5812	1097040	99880	3080
12M	97.61%	98.10%	97.88%	97.83%	1.2112*e*−06	1.76633*e*−05	0.3709	0.3	0.5	0.7	2194369	193978	11653	2202570	191200	6230
16M	97.10%	97.95%	97.31%	97.31%	1.3585*e*−06	5.4527*e*−05	0.4107	0.3	0.5	0.7	2989587	197848	12565	3000709	192848	6443
20M	98.14%	99.25%	98.21%	98.34%	1.0944*e*−06	4.2757*e*−06	0.3975	0.3	0.5	0.7	3758357	226954	14689	3800294	192894	6812
24M	96.3%	96.90%	96.37%	96.37%	1.0512*e*−06	2.5594*e*−06	0.3868	0.3	0.5	0.7	4430140	347239	22621	4557555	235331	7114
28M	98.12%	99.30%	98.34%	98.34%	1.0072*e*−06	9.7398*e*−06	0.3884	0.3	0.5	0.7	5187872	385435	26693	5337235	254157	8608
30M	96.48%	98.21%	97.87%	98.04%	1.9847*e*−06	0.3458	0.5027	0.3	0.5	0.7	5504412	465781	29807	5705847	284278	9875

The observed variation in performance across different dataset sizes is primarily influenced by differences in data distribution, class balance, and sample representation. Smaller datasets may exhibit higher variability due to limited samples, while larger datasets provide more stable and diverse patterns, enabling better generalization. In addition, the proposed framework relies on probability-based thresholding and clustering mechanisms, which become more effective as the dataset size increases. This leads to improved separation of risk categories and more consistent authentication decisions for larger datasets.

### 4.4 Ablation study of the proposed framework

An ablation study is performed to understand the role of each component in the proposed framework. The ensemble model provides high classification performance, while the additional modules improve authentication decisions based on predicted probabilities. ROC-based thresholding provides an initial decision boundary, but it results in a high number of additional authentication requests and denied logins. Clustering improves this by generating adaptive thresholds from the data, leading to a better distribution of decisions. Fuzzy logic handles uncertainty by enabling gradual transitions between risk levels instead of rigid decisions. Finally, L-BFGS-B optimization adjusts the thresholds to achieve a better balance between security and usability.

The results show that each component contributes to improving the quality of authentication decisions, particularly by reducing unnecessary authentication requests and minimizing false denials (Table 7).

**Table 7:** Ablation study of the proposed framework.

Configuration	Accuracy	Allow	Req.Auth	Deny	Key Contribution
Ensemble (GB + SVM + XGB)	97.77	–	–	–	Baseline classification model
+ ROC Threshold	97.77	89,798	208,335	101,867	Static threshold causes high authentication and denial
+ ROC + Clustering	97.77	349,432	48,132	2,436	Adaptive thresholds improve decision distribution
+ Fuzzy Logic	97.77	–	–	–	Enables gradual risk transitions
+ Optimization (L-BFGS-B)	97.77	349,923	48,849	1,228	Optimizes balance between security and usability

“–” indicates that the metric is not applicable at that stage, as action-level decisions are generated only after thresholding.

### 4.5 Adversarial robustness analysis

In real-world authentication systems, input data may be affected by noise, missing values, or potential manipulation. Therefore, it is important to examine how the proposed model behaves under such conditions. Robustness in this context refers to the ability of the model to maintain reasonably stable performance when the input data is slightly altered. To evaluate this, controlled perturbations were introduced into the dataset, including Gaussian noise, missing feature values, and simulated attack scenarios. Gaussian noise with zero mean (μ = 0) was applied to ensure unbiased perturbation without shifting the original data distribution. Missing data was simulated by randomly masking a portion of the features, while simulated attack scenarios were created by scaling feature values to represent abnormal login behavior.

The results are presented in Table 8. The model achieves a baseline accuracy of 97.77% on normal data. When small perturbations are introduced (σ = 0.05), only a slight decrease in performance is observed. With increased perturbation (σ = 0.10), the performance decreases moderately but remains acceptable. In the case of incomplete data, the model continues to perform well, indicating its ability to handle missing information. Under simulated attack conditions, the model maintains reasonable performance, suggesting its capability to detect abnormal patterns. Overall, the results indicate that the proposed model is reasonably stable under different perturbation conditions, making it suitable for practical authentication scenarios.

**Table 8:** Robustness analysis under perturbed and simulated attack conditions.

Scenario	Perturbation Parameters	Accuracy (%)
Normal Data	No perturbation	97.77
Feature Perturbation (σ=0.05)	Gaussian noise (μ=0, σ=0.05)	96.12
Feature Perturbation (σ=0.10)	Gaussian noise (μ=0, σ=0.10)	94.68
Incomplete Data	Random masking (10% features set to 0)	95.21
Simulated Attack Scenario	Feature scaling (×1.1–1.3 random)	93.87

## 5 Model explainability

Artificial intelligence (AI) algorithms are widely used to assist users in decision-making. However, people may not fully understand how AI reaches its conclusions or the reasoning behind its outputs. This lack of clarity may hinder users’ ability to trust or interpret the results. To address this issue, explainable artificial intelligence (XAI) has been developed. XAI is a technology designed to make the processes of AI models more visible to users. It does this by providing detailed explanations of the methods used, the entire process, and the outcomes of the AI in a simple manner, that is easy for the user to understand [8].

Model explainability in risk-based authentication increases transparency and assure authentication decision-making. Global and local explainability methods were combined in this study to improve the interpretability of the ensemble model. The global approach of explainability identifies the main risk factors that affect the authentication process and thus sets the direction for security policies. In contrast, local explainability examines specific login attempts to help administrators identify false positives and negatives [16]. The SHAP methodology exhibits feature importance through TreeExplainer for the first 5,000 sample instances. The ensemble model not only turns out to be highly precise but also interpretable and trustworthy, hence, top-notch decision-making in security-sensitive areas. The SHAP research advocates that the security team should clarify authentication policies by revealing the logic behind the model’s decisions. For example, when the system observes a large amount of genuine traffic coming from a specific location being classified as very risky, it might require a second factor of authentication rather than blocking access. If the type of device is one of the main aspects that the model relies on, device identification can be refined. This approach not only improves security, precision, and adaptability but also reduces the number of misidentifications of legitimate users and maintains the safety of accounts.

### 5.1 Local explainability

**Fig 10:**
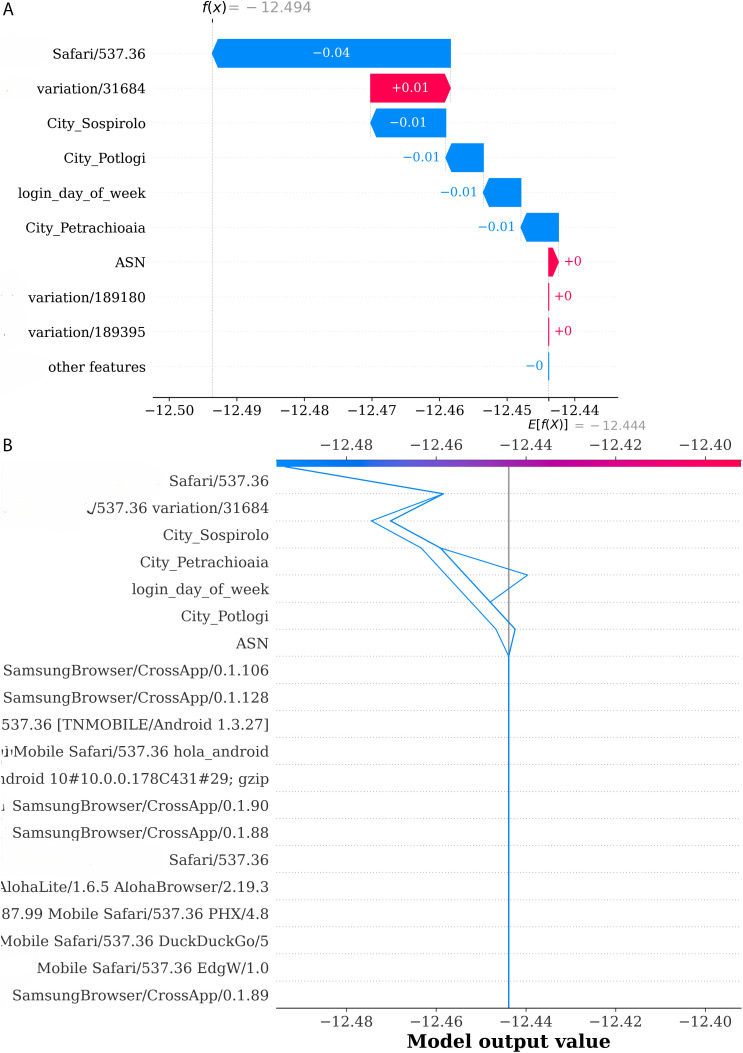
Local explainability of the model is shown in (a) with the Waterfall Plot for the first instance and in (b) with the Decision Plot for the first 10 instances, displaying feature contributions to the predictions.

Different methods have been applied to explain the predictions at the individual level. Here are waterfalls and decision plots to show the model’s behavior clearer for particular cases. The SHAP waterfall plot in (Fig 10a) illustrates the breakdown of every feature’s contribution to the model’s prediction for a single instance. The model’s base value is −12.494, which indicates the expected output before adding any feature contributions. The User Agent String feature accounts for −0.04, thus reducing the model’s prediction, where City Sossiprolo and City Potlogi each account for −0.01, which is only a minor decrease in the prediction. The login_day_of_week feature accounts for −0.01, which means that logins on this specific day have a slight negative impact on the event being classified as account takeover. In the same way, Petrachioia and ASN have a negative contribution of −0.01, thus making the model prediction even less likely. The final prediction after summing all feature contributions is −12.444, which indicate that this specific login attempt is categorized as not suspicious (normal). This breakdown shows that although the contribution of each feature may be small, it is still significant in determining the model output. The SHAP decision plot presented in (Fig 10b) has a starting point of −12.494, which indicates the average model output before any feature is considered. As different features contribute SHAP values, the blue line reflects the overall impact of these contributions. The user-agent string feature has a negative contribution, slightly lowering the prediction and suggesting a normal login. Similarly, the characteristics of the cities, Sossiprolo, Potlogi, and Petrachioia, also contribute negatively, further reducing the prediction. However, the Login Day of Week feature contributes positively, slightly increasing the prediction for a normal login. Finally, the ASN has a small negative contribution, that decreases the likelihood of an account takeover. The final prediction value of −12.44 indicates that the log-in is classified as non-suspicious.

### 5.2 Global explainability

**Fig 11:**
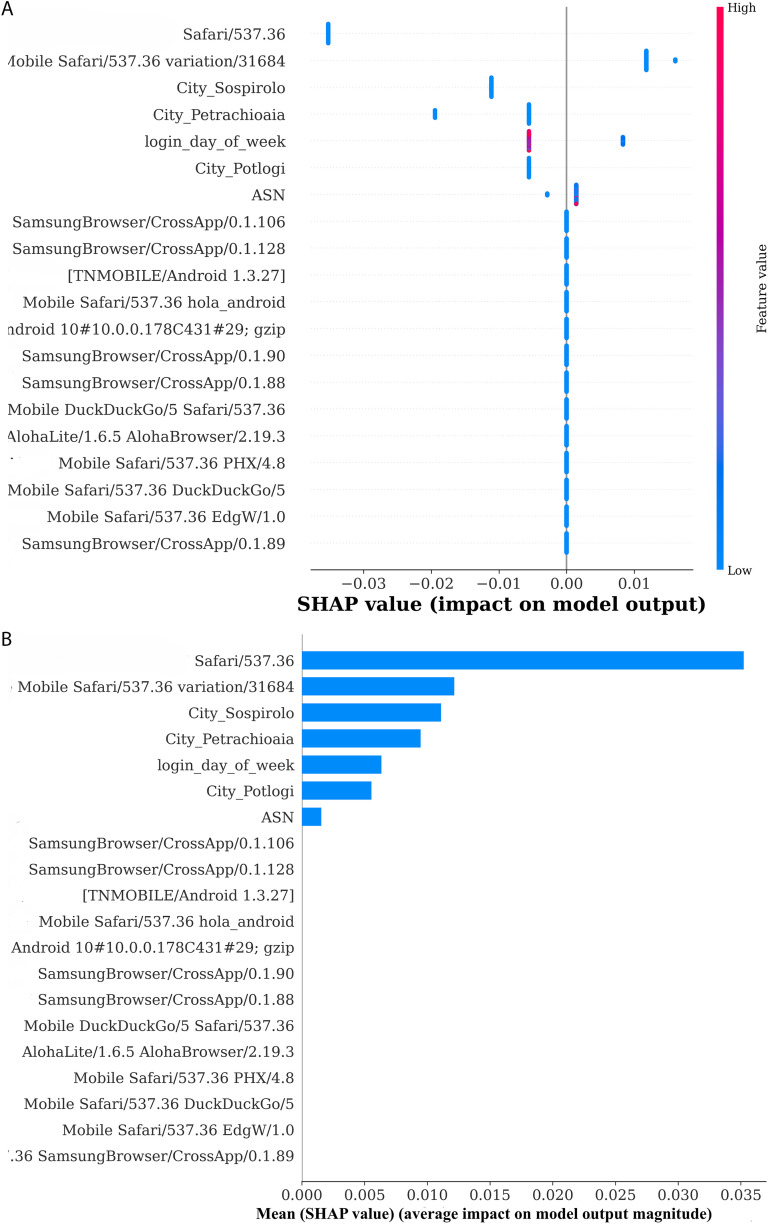
Global explainability of the developed model is depicted in (a) through the Summary Plot, while (b) presents the Bar Plot showing the overall feature importance.

As illustrated in (Fig 11a), the SHAP summary plot provides the most informative detailed picture of the importance of the features, along with their performance snippets on the model’s decision. The SHAP values displayed on the X-axis, representing how much each feature contributes to the model’s output, are the main points of the axis. Positive values on the Y-axis indicate a push of the prediction higher, and negative values indicate a lowering of the prediction. The Y-axis portrays the features according to their overall importance from bottom to top. The features at the top have a greater impact on the prediction than those at the bottom. The color gradient depicts the feature value, with blue signifying lower values and red denoting higher values. The feature string of the user-agent impacts the prediction of the model dually, that is, with both positive and negative contributions that vary according to the instance. The prediction is also affected by less impactful, but still important, other features such as city ossiprolo, city potlogi, ASN, and login_day_of_week. Thus, this plot provides a picture of the most influential features and how the respective values skylark the model’s decisions. (Fig 11b) shows an SHAP bar plot where the user-agent string is the feature with the most significant positive impact on the model with a mean SHAP value of approximately 0.035, which indicates that it greatly influences the prediction of normal logins. Sossiprolo, Potlogi, and Petrachioia were the three cities that contributed positively, each with a mean SHAP value of approximately 0.01. The login_day_of_week and ASN features also had small positive impacts, which were reflected in mean SHAP values of 0.005 and 0.005, respectively, implying that they slightly favored normal logins. To summarize, the user agent string was the most significant feature, whereas the city-related and time-based features showed a moderate influence overall.

## 6 Conclusion

In conclusion, the research concluded that sophisticated machine learning techniques are the best methods for detecting account takeovers when employing RBA. Several algorithms were evaluated, including ensemble, gradient boosting, random forest, SVM, and XGBoost. The ensemble model always led, achieving an accuracy of 97.77%, precision of 99.41%, recall of 98.04% and an F1-score of 98.72%. The risk thresholds were modeled and optimized using the ROC-based and ROC + clustering methods. The low, medium, and high risk categories were initially set at 1.9601*e*^−06^, 0.3371, and 3.7066*e*^−06^, respectively. After employing ROC + Clustering and further tuning via L-BFGS-B optimization, the thresholds were adjusted to 0.3, 0.5, and 0.7, respectively. This optimization considerably contributes to the accuracy of the decision-making process.

The performance after optimization shows clear improvement in managing the authentication process. For the 2M dataset, the number of Allow Login actions increased slightly from 349,432–349,923, while Deny Login actions decreased from 1,462–1,228. In addition, the number of additional authentication requests showed a slight reduction from 49,106–48,849. Similar trends are observed for larger datasets, including the 30M dataset, demonstrating the scalability and effectiveness of the proposed framework. The incorporation of fuzzy logic further enhanced the system by enabling dynamic adjustment of security levels, allowing smoother transitions between risk categories. Furthermore, explainable AI (XAI) techniques, particularly SHAP, were utilized to improve the transparency and interpretability of the model’s decision-making process, thereby increasing trust and understanding of the predictions. The model consistently achieves high accuracy across different dataset sizes, demonstrating its scalability and effectiveness for real-world risk-based authentication (RBA) applications.

Overall, the proposed work shows significant improvement in the field of risk-based authentication. The developed machine learning models enhance both security and user experience. In particular, the ensemble model, combined with threshold optimization, fuzzy logic, and explainable AI, achieves high accuracy and improves decision-making. The model also performs consistently across different dataset sizes, demonstrating its scalability and practical applicability in real-world scenarios. In addition, the use of real-time data, detailed risk factors, and time-based features further improves prediction accuracy. For future work, the development of a lightweight and computationally efficient model will be considered to reduce processing time and improve real-time performance. Further enhancements such as continuous learning, advanced fuzzy logic, and integration with multi-factor authentication (MFA) can provide more robust risk scoring and layered security without affecting user experience.
